# Ultra-processed foods consumption and diet quality among preschool children and women of reproductive age from Argentina

**DOI:** 10.1017/S1368980022002543

**Published:** 2023-11

**Authors:** María Elisa Zapata, Gustavo Cediel, Ezequiel Arrieta, Alicia Rovirosa, Esteban Carmuega, Carlos A Monteiro

**Affiliations:** 1 Center of Studies in Child Nutrition Dr. Alejandro O´Donnell (CESNI), Cerrito 1136 1° Floor, Ciudad Autónoma de Buenos Aires, 1010, Argentina; 2 University of Antioquia, School of Nutrition and Dietetics, Medellín, Antioquia, Colombia; 3 Multidisciplinary Institute of Plant Biology (IMBIV), CONICET and National University of Cordoba, Córdoba, Argentina; 4 Center for Epidemiological Research in Nutrition and Health, University of São Paulo, São Paulo, Brazil

**Keywords:** Food processing, Ultra-processed food, Nutrients, Healthy diet, Non-communicable diseases

## Abstract

**Objective:**

To assess the association between the consumption of ultra-processed foods (UPF) and diet quality among preschool children and women of reproductive age from Argentina.

**Design:**

Cross-sectional and nationally representative survey. The food items were classified according to the NOVA system. Consumption of fruits, vegetables, legumes, nuts, seeds and wholegrains was estimated, and the energy and nutrients related to non-communicable disease (NCD) intake. Linear regression was used to assess the associations.

**Setting:**

Argentina.

**Participants:**

Children aged 2–5 years (*n* 7022), female adolescent aged 10–19 years (*n* 2165) and women aged 20–49 years (*n* 4414).

**Results:**

UPF represented more than a quarter of total energy intake, 27 % in children, 31 % in female adolescents and 26 % in women. Across all age groups, the major contributors to UPF consumption were cookies and pastries (about 6·0–7·0 %), soft drinks (about 2·7–3·7 %), candies (about 1·8–4·6 %), and juices (about 1·3–1·7 %). The consumption of fresh vegetables, fresh fruits and legumes was negatively associated with UPF consumption. A significant positive association was found between the dietary share of UPF and the dietary content of NCD-promoting nutrients such as free sugars and total saturated and *trans*-fats. In contrast, a significant negative association was found with the content of NCD-protective such as fibre and protein.

**Conclusions:**

UPF were associated with lower consumption of healthy foods and higher intake of nutrients related to NCD in children and women of reproductive age in Argentina. It is necessary to design food policies that simultaneously reduce the consumption of UPF while promoting the intake of fresh and whole foods to improve the dietary quality.

Diet is a significant determinant of human health^(1)^, and the relationship between food consumption and non-communicable diseases (NCD) has been extensively reported^(2)^. According to the Global Burden of Disease Study, one in five deaths and one in six disability-adjusted life years are attributed to poor-quality diets, representing the most critical risk factor globally^(3,4)^. Projections indicate that food-related diseases’ prevalence will increase in the following decades unless strategies to modify the trajectory are applied, particularly in low- and middle-income countries^(5)^.

Ultra-processed foods (UPF) are industrial formulations manufactured with multiple ingredients, typically containing cosmetic additives such as colourings, flavourings, sweeteners and emulsifiers, with little whole food. Because UPF are typically ready-to-consume, cheap, hyper-palatable, and aggressively marketed food and drink products^(6)^, their sales and consumption have increased progressively and steadily over the last decades globally^(7)^. UPF are energy-dense and highly concentrated in unhealthy nutrients such as Na, free sugars and unhealthy fats (*trans* and saturated fats)^(8)^.

The characteristics mentioned above make UPF highly preferred by consumers, generating a displacement effect in consuming fresh or minimally processed foods such as fruits, vegetables and legumes. Several studies have shown that UPF have displaced and are displacing staple foods worldwide, shaping the food supply, the food culture and the dietary patterns^(9)^. The nature of UPF indicates that these changes are harmful to public health^(10)^. Several studies based on nationally representative cross-sectional surveys have shown that increased UPF intake is associated with higher content of nutrients related to NCD and decreasing the dietary diversity of natural foods and the content of fibre and vitamins^(11–18)^. In recent years, several studies revealed associations of high consumption of UPF with several NCD such as for overweight and obesity^(19,20)^, type 2 diabetes^(21)^, hypertension^(22)^, dyslipidemias^(23)^, coronary diseases, stroke^(24)^, metabolic syndrome^(19,20)^, all cancers and breast cancer^(25)^.

Argentina is a middle-income country with malnutrition in all its forms but has comparatively lower undernutrition indices and higher overweight prevalence^(26)^ than the other countries in Latin America^(3)^. The prevalence of obesity reaches 3·6 % in <5 years, 20·4 % in the population aged 5 to 17 years, and 33·9 % in the population 18 old years and over^(27)^, 12·3 % of adults report high blood glucose or diabetes, 28·9 % high cholesterol and 34·7 % high blood pressure^(28)^. Remarkably, Argentina has one of the region’s highest UPF sales per capita^(29)^. Despite the relevance of the issue, few studies analysed the contribution of UPF in diets^(30,31)^, but it was limited to the assessment of nutrients related to NCD. Therefore, the present study aims to assess the influence of UPF consumption on the intake of specific food groups and critical nutrients for developing NCD in the Argentinian population of children and women of reproductive age, using dietary data collected from a nationally representative nutritional survey.

## Methods

### Data source and sampling

We utilised dietary data collected during the 2005 National Survey of Health and Nutrition (ENNyS) of Argentina, a nationally representative and cross-sectional survey of maternal and child populations of urban areas carried out by the Ministry of Health^(32)^. Using a probabilistic multi-stage sample involving localities, census blocks and three independent samples: 32 474 boys and girls between 6 months and 5 years, 8307 women aged 11–49 years, and 1941 pregnant women. We analysed data from 7022 children (2–5 years of age), 2165 adolescent females (10–19 years of age) and 4414 adult women (20–49 years of age).

Food consumption was collected at the interviewee’s home by one 24-h dietary recall interview conducted by trained nutritionists, for adolescents and adults or to the person responsible for feeding young children in the case of 2–5-year-old participants. The institution’s managers gave information about the food consumed in day-care centres and schools by children and adolescents. It was registered the consumption of everything eaten by individuals the day before the survey, including food, beverages (except drinking water and infusions), and mineral and vitamin supplements of particular interest. The interviewers used visual food models with colour photographs of portions of different food sizes and references of raw and cooked weight amounts. The composition of some preparations was previously standardised using essential and regional recipes. Details on the methodology of ENNyS can be found elsewhere^(32)^.

Additional information for the analysis was obtained from a general questionnaire with details of household characteristics and assets and sociodemographic characteristics of the head of households and participants. Aspects of the study were provided, and participants were asked to sign informed consent before their inclusion in the study.

### Food classification according to processing

The food items were sorted into mutually exclusive food subgroups within: (1) unprocessed or minimally processed foods (eleven subgroups: e.g. fresh meat, roots, and tubers, cereals, vegetables, legumes, fruits); (2) processed culinary ingredients (four subgroups: e.g. plant oils, table sugar, animal fats); (3) processed foods (five subgroups: e.g. unpackaged fresh bread, cheese, ham, and salted meat, vegetables and fruits preserved in brine or sugar syrup); and (4) UPF (seventeen subgroups: e.g. carbonated soft drinks, sweet or savoury snacks, confectionery, industrial desserts, reconstituted meat products, shelf-stable or frozen meals, industrial packaged bread), according to the grade of processing by following the NOVA system classification^(6)^. Details of categorisation can be found in Table 1 in Supplemental Material.

### Total energy and nutrient intake assessment

To obtain the nutritional profile of diets, we estimated the energy and nutrients provided by each food item. Given that a complete local database for the nutrient compositions of foods and beverages is not available for Argentina, we combined different sources of nutritional composition databases. Although the Argenfoods database^(33)^ was the primary source, we also utilised the USDA database^(34)^, the Germany database^(35)^ and the National Nutritional Institute of Salta University database^(36)^. Also, we have used the information from food labels for some products that are not reported in any of the databases mentioned above and some data obtained in the CESNI laboratory (*Centro de Estudios Sobre Nutrición Infantil, a Non-Government Organization of Argentina*).

Thus, the diet’s nutrient composition was assessed by the daily intake of energy, total proteins, total carbohydrates, available carbohydrates, free sugars, fibre, total fats, saturated fats, Na and K. It was calculated considering the edible part of each food item and their energy and nutrient composition. Finally, the contribution of every NOVA food group was estimated.

### Data analysis

The intake was reported (mean and standard error) in absolute terms (grams or milligrams) and considering the energy intake relative to the total daily intake of the specific participant.

The dietary pattern of children, adolescent females and adult females was described by distributing the total energy intake according to the four NOVA food groups, and within these groups, according to selected subgroups.

After that, we analysed the average energy contribution of UPF and the impact of the consumption of these products on specific food groups and critical nutrients related to NCD by considering the recommendations of the WHO^(37)^. Firstly, the 421 food items reported in the 24-h dietary recall were classified into five food groups, fresh vegetables, fresh fruits, legumes, nuts and seeds, and wholegrains (Table 2 in Supplemental Material) and were estimated the consumption in 2000 kcal/d. Secondly, we assess the daily intake of protein, free sugar, fibre, total fats, saturated fat, *trans*-fat, fibre, Na and K^(37–41)^. Fibre, Na and K intake were expressed per 2000 kcal, while the other nutrients were expressed as a percentage of total energy intake. The energy density of solid fraction was calculated by dividing the sum of energy from the intake of solid foods by the amount in grams of these foods. The recommendations used for this indicator were those proposed by the World Cancer Research Fund^(42)^.

Each nutritional indicator was estimated for the overall diet, for NOVA 1-2-3 categories (the fraction of non-UPF) and NOVA 4 category (the fraction of UPF). The indicators were used to evaluate the dietary quality of the population strata corresponding to the distribution quintiles of caloric contribution from UPF to total calories. Linear regression analyses were used to identify the direction and the statistical significance of the association between the distribution quintiles of caloric contribution from UPF (as % of the energy of UPF) and nutritional indicators as the dependent variable, with and without fitting for confounding variables (age, gender in children 2–5 years, geographic region, years of schooling of the head of the family in children and adolescent aged 10–19 years, years of education of women aged 20–49 years, unsatisfaction of basic needs, household income per capita divided into quintiles).

Individuals were classified into five strata according to the caloric value that UPF contributed to the total value of their diet. These strata were related to the distribution quintiles of caloric contribution from UPF across the population.

## Results

### Distribution of total energy intake by food group

**Fig. 1:**
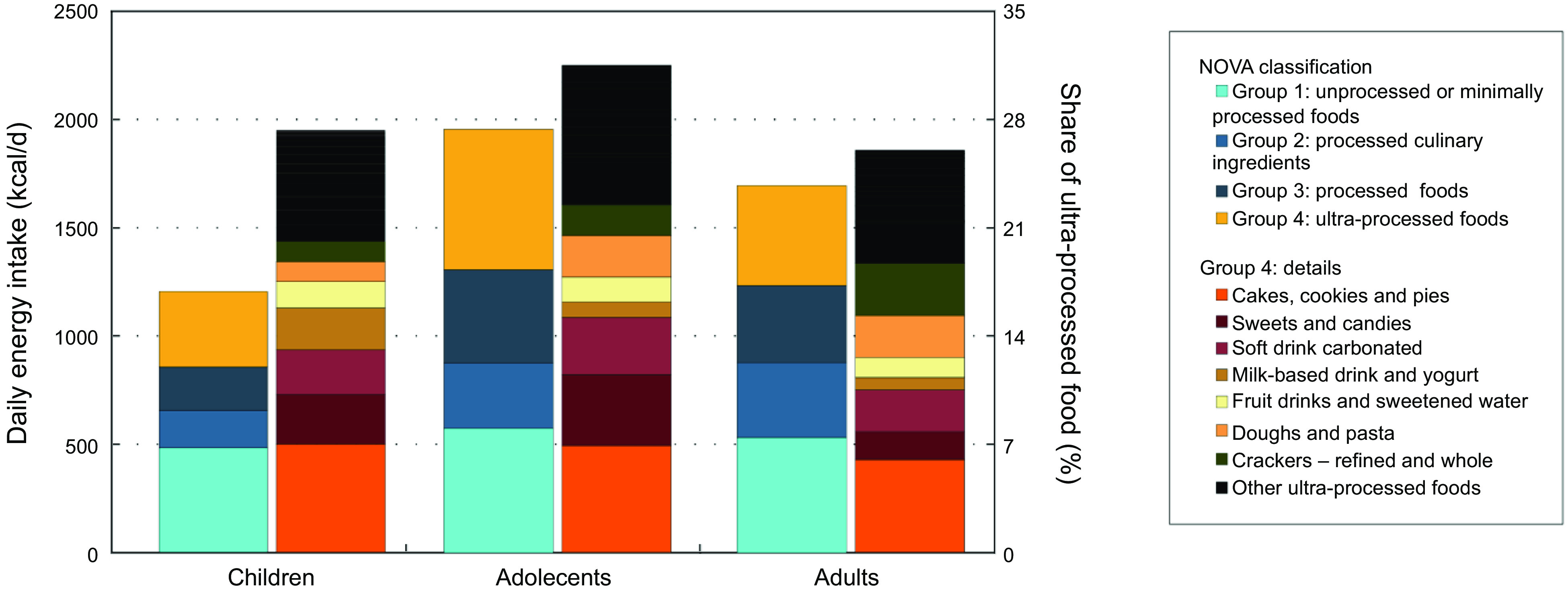
Contribution to the daily energy intake of each NOVA food group (first bar) and share of total energy of ultra-processed food (second bar). Argentinian children and women population (2005).

We estimated a mean daily energy intake of 1636 ± 620 kcal among children, 1956 ± 829 kcal in adolescent females and 1695 ± 800 kcal in adult females, with differences in the participation of each NOVA food group (Fig. 1). For more details on the contribution of each food item, see Table 1.

**Table 1:** Distribution of total energy intake according to NOVA food groups. Argentinian children and women population (2005)

NOVA food groups	Children (2–5 years of age) *n* 7022				Adolescent (10–19 years of age) *n* 2165				Women (20–49 years of age) *n* 4414			
	Absolute (kcal/d)		Relative (% of total energy intake)		Absolute (kcal/d)		Relative (% of total energy intake)		Absolute (kcal/d)		Relative (% of total energy intake)	
	Mean	se	Mean	se	Mean	se	Mean	se	Mean	se	Mean	se
Group 1: Unprocessed or minimally processed foods	653·3	3·6	41·3	0·2	574·8	6·9	31·2	0·3	530·9	5·2	33·1	0·3
Milk and plain yogurt	235·6	2·3	15·1	0·1	68·8	2·1	3·7	0·1	45·4	1·2	2·9	0·1
Meat	156·2	1·7	9·7	0·1	199·4	4·0	10·7	0·2	201·1	3·1	12·3	0·2
Cereals	135·7	1·9	8·6	0·1	169·4	4·4	9·1	0·2	150·5	2·8	9·1	0·2
Fruits	48·2	0·9	3·0	0·1	38·4	1·5	2·2	0·1	38·7	1·0	2·6	0·1
Roots and tubers	43·4	0·8	2·8	0·0	48·5	1·6	2·7	0·1	41·7	1·0	2·7	0·1
Egg	18·8	0·4	1·1	0·0	26·5	0·9	1·4	0·0	26·0	0·7	1·5	0·0
Vegetables	10·7	0·2	0·7	0·0	15·0	0·5	0·9	0·0	18·9	0·4	1·4	0·0
Legumes	3·4	0·3	0·2	0·0	4·7	0·7	0·3	0·0	4·6	0·5	0·3	0·0
Fish and seafood	1·0	0·1	0·1	0·0	1·1	0·3	0·1	0·0	3·0	0·4	0·2	0·0
Nuts	0·3	0·1	0·0	0·0	3·0	0·6	0·1	0·0	1·0	0·2	0·0	0·0
Group 2: Processed culinary ingredients	235·9	1·8	14·8	0·1	300·4	4·2	16·0	0·2	345·3	4·1	20·5	0·2
Table sugar	107·3	1·0	7·1	0·1	132·9	2·7	7·3	0·2	172·9	3·1	10·5	0·2
Plant oils	99·5	1·2	6·1	0·1	134·4	2·7	7·1	0·1	135·7	2·1	8·1	0·1
Animal fats	27·6	0·8	1·6	0·0	31·7	1·7	1·4	0·1	34·5	1·2	1·7	0·1
Other processed culinary ingredients	1·5	0·2	0·1	0·0	1·5	0·3	0·1	0·0	2·2	0·3	0·1	0·0
Group 3: Processed foods	272·2	3·1	16·4	0·2	431·4	8·1	21·3	0·3	357·4	5·1	20·5	0·2
Fresh unpackaged bread	196·7	2·7	12·3	0·2	299·0	6·7	15·2	0·3	220·3	4·0	13·0	0·2
Cheese	29·5	0·8	1·7	0·0	59·5	2·4	2·8	0·1	64·3	1·7	3·5	0·1
Ham and other salted, smoked or canned meat or fish	17·5	0·9	0·9	0·0	28·3	2·0	1·3	0·1	27·8	1·6	1·4	0·1
Vegetables, fruits and other plant foods preserved in brine or syrup	3·7	0·1	0·2	0·0	5·4	0·4	0·3	0·0	5·0	0·2	0·3	0·0
Fermented alcoholic beverages	0·0	0·0	0·0	0·0	0·8	0·2	0·0	0·0	11·2	0·9	0·6	0·0
Other processed foods	24·7	1·0	1·4	0·1	38·4	2·7	1·8	0·1	28·9	1·5	1·7	0·1
Group 4: Ultra-processed foods	474·4	4·9	27·5	0·2	648·9	11·8	31·5	0·4	461·6	6·6	25·9	0·3
Cakes, cookies and pies	124·7	2·3	7·0	0·1	148·6	5·5	6·9	0·2	113·3	3·3	6·0	0·2
Sweets and candies	57·4	1·4	3·2	0·1	100·0	4·1	4·6	0·2	32·8	1·6	1·8	0·1
Soft drinks carbonated	49·6	1·1	2·9	0·1	77·8	2·8	3·7	0·1	49·6	1·5	2·7	0·1
Milk-based drink and yogurt	41·8	1·1	2·7	0·1	17·0	1·2	1·0	0·1	11·6	0·6	0·8	0·0
Fruit drinks and sweetened water	26·5	0·6	1·7	0·0	28·7	1·3	1·6	0·1	20·4	0·8	1·3	0·0
Doughs and pasta	25·3	1·2	1·3	0·1	54·3	3·8	2·7	0·2	51·6	2·4	2·7	0·1
Crackers – refined and whole	20·4	0·8	1·3	0·0	35·9	2·1	2·0	0·1	51·4	1·6	3·4	0·1
Bread packaged	20·6	0·9	1·1	0·0	51·7	3·0	2·7	0·2	44·3	1·8	2·6	0·1
Cocoa and milk flavourings	15·1	0·4	0·9	0·0	8·3	0·6	0·4	0·0	1·6	0·2	0·1	0·0
Milk desserts	14·6	0·7	0·9	0·0	3·4	0·6	0·2	0·0	1·8	0·3	0·1	0·0
Reconstituted meat	15·9	0·9	0·8	0·0	26·4	1·9	1·2	0·1	16·0	1·0	0·9	0·1
Desserts	11·9	0·7	0·7	0·0	10·5	1·3	0·5	0·1	11·4	0·9	0·6	0·0
Salty snacks	11·1	0·6	0·6	0·0	20·6	2·0	0·8	0·1	6·2	0·8	0·3	0·0
Breakfast cereals and cereal bars	10·4	0·6	0·6	0·0	5·7	0·7	0·3	0·0	4·8	0·4	0·3	0·0
Sauces, dressings and gravies	9·7	0·5	0·5	0·0	30·7	2·0	1·4	0·1	16·1	0·9	0·8	0·0
*“Dulce de leche”*	8·0	0·4	0·4	0·0	10·0	0·9	0·4	0·0	8·0	0·6	0·4	0·0
Sandwiches and hamburgers on bun	5·9	0·4	0·3	0·0	14·7	1·4	0·9	0·1	9·3	0·9	0·5	0·1
Infant formula	1·3	0·3	0·1	0·0								
Other non-alcoholic beverages	1·1	0·1	0·1	0·0	0·6	0·2	0·0	0·0	3·2	0·4	0·2	0·0
Cheese – spreadable and melted	1·1	0·1	0·1	0·0	1·8	0·3	0·1	0·0	4·8	0·4	0·3	0·0
Other ultra-processed foods	2·0	0·2	0·1	0·0	2·1	0·4	0·1	0·0	3·4	0·3	0·2	0·0

Other processed culinary ingredients include cornstarch and honey. Other processed foods include salted roasted peanuts, *chips, churros*, potato gnocchi, fried cakes **(** *torta fritas), dulce de batata, dulce de membrillo, jalea de membrillo* and fruit jam. Other ultra-processed foods include creamy canned maize, canned diet peaches, canned diet pears, instant and canned soups, instant meals, margarine, and distilled alcoholic beverages.

Minimally processed food accounted for 41 % of daily energy intake in children, while in adolescent and adult females, the intake of minimally processed foods provided 31 % and 33 % of daily energy, respectively. Milk and plain yogurt were the essential food items in children (15 %), and meat and cereals contributed with most calories in adolescent and adult females (10–12 %). The relative energy intake from processed culinary ingredients was 15 % in children, 16 % in adolescent females and 20 % in adult females, being table sugar the primary source of calories in this group (about 7–10 %). Processed foods contributed 16 % of total calories in children, 21 % in adolescent females and 20 % in adult females. The main contributor was fresh bread with about 12–15 % of total energy.

Concerning UPF, they represented 27·5 % of daily energy in children and 31·5 % in adolescent females, while in adult females, the participation was 26 %. Most calories from UPF were related to cookies, pastries, cakes, packaged bread and crackers (about 11 % of total daily intake), followed by soft drinks and juices (about 5 % of total daily intake). Sweets and candies represented 5 % of total daily intake in children and adolescents and 2 % in adult women. Milk-based drinks and yogurt contributed 3 % of energy in children and 1 % in adolescents and women.

### The nutrient profile of the diet

As can be observed in Table 2, the overall diet of the analysed Argentinian population presented an unhealthy profile. On the one hand, the number of fruits and vegetables consumed was very low (169–227 g/d), well below the WHO recommendations (400 g/d). In addition, the total intake of wholegrains, legumes, nuts and seeds reached a low quantity of 7–14 g/d. On the other hand, the consumption of free sugar and saturated fats exceeds the recommended levels (<10 % of the total energy of both nutrients) in the three age groups. Moreover, the consumption of fibre and potassium was deficient, three- and twofold below the optimal intake level, respectively. In addition, although the reported Na intake was relatively low, the Na:K ratio was <1:3 in all groups. However, the survey did not account for salt intake during meal preparation or at the table. Thus, Na consumption could have been higher than the figure reported here, increasing the Na:K ratio further. It is worth mentioning that the consumption of *trans*-fats was just below the recommended intake (<1 %).

**Table 2:** Indicators of diet quality of the overall diet and two diet fractions. Argentinian children and women population (2005)

Indicators	Children aged 2–5 years						Adolescent aged 10–19 years						Women aged 20–49 years					
	Overall diet (*n* 7022)		Fraction of the diet made up of non-ultra-processed foods (*n* 7017)		Fraction of the diet made up of ultra-processed foods (*n* 6537)		Overall diet (*n* 2165)		Fraction of the diet made up of non-ultra-processed foods (*n* 2164)		Fraction of the diet made up of ultra-processed foods (*n* 1973)		Overall diet (*n* 4414)		Fraction of the diet made up of non-ultra-processed foods (*n* 4413)		Fraction of the diet made up of ultra-processed foods (*n* 3918)	
	Mean	se	Mean	se	Mean	se	Mean	se	Mean	se	Mean	se	Mean	se	Mean	se	Mean	se
Fresh vegetables (g/2000 kcal)	65·4	1·0	65·4	1·0	0·0	0·0	84·6	2·8	84·6	2·8	0·0	0·0	126·1	3·9	126·1	3·9	0·0	0·0
Fresh fruits (g/2000 kcal)	104·8	1·9	104·8	1·9	0·0	0·0	85·9	3·8	85·9	3·8	0·0	0·0	100·8	2·9	100·8	2·9	0·0	0·0
Legumes (g/2000 kcal)	4·1	0·3	4·1	0·3	0·0	0·0	4·7	0·5	4·7	0·5	0·0	0·0	6·5	0·5	6·5	0·5	0·0	0·0
Nuts and seeds (g/2000 kcal)	0·2	0·0	0·2	0·0	0·0	0·0	1·0	0·1	1·0	0·1	0·0	0·0	0·5	0·1	0·5	0·1	0·0	0·0
Wholegrains (g/2000 kcal)	2·8	0·2	2·9	0·3	1·4	0·2	2·3	0·2	1·6	0·3	4·0	0·8	6·7	0·4	2·9	0·4	15·7	1·0
% of total energy intake from:			72·5	0·2	27·5	0·2			68·5	0·4	31·5	0·4			74·1	0·3	25·9	0·3
Total protein	14·3	0·0	17·0	0·1	8·5	0·1	13·7	0·1	16·7	0·1	7·8	0·2	14·4	0·1	16·7	0·1	9·7	0·2
Total carbohydrates	56·4	0·1	51·5	0·2	69·1	0·2	57·0	0·3	53·0	0·4	65·7	0·4	55·4	0·2	52·1	0·3	64·9	0·3
Available carbohydrates	54·4	0·1	49·2	0·2	68·0	0·2	54·6	0·2	50·3	0·4	64·2	0·4	52·9	0·2	49·4	0·3	62·9	0·3
Free sugar	18·0	0·1	10·3	0·1	42·8	0·3	18·0	0·2	11·6	0·2	37·8	0·6	18·0	0·2	14·8	0·2	31·6	0·4
Total fat	31·3	0·1	33·9	0·1	23·4	0·2	31·7	0·2	33·0	0·3	27·9	0·4	32·2	0·2	33·3	0·2	27·3	0·3
Saturated fat	12·1	0·0	13·4	0·1	8·7	0·1	10·4	0·1	10·9	0·1	9·1	0·1	10·6	0·1	10·9	0·1	9·1	0·1
*Trans*-fat	1·0	0·0	0·8	0·0	1·2	0·0	0·9	0·0	0·6	0·0	1·3	0·0	0·9	0·0	0·6	0·0	1·3	0·0
Na density (mg/2000 kcal)	1321	8	1154	7	6083	412	1354	17	1225	19	4060	510	1390	14	1177	13	6209	506
K density (mg/2000 kcal)	2429	9	2917	12	1401	17	1984	18	2436	24	1148	28	2105	17	2526	22	1238	24
Na:K ratio	0·6	0·0	0·4	0·0	3·7	0·1	0·9	0·0	0·6	0·0	3·8	0·2	0·8	0·0	0·6	0·0	4·8	0·1
Fibre density (g/2000 kcal)	10·2	0·1	11·6	0·1	5·7	0·1	11·9	0·1	13·3	0·2	7·5	0·2	13·0	0·2	13·7	0·2	10·0	0·2
Energy density of solid foods (kcal/g)	2·1	0·0	1·9	0·0	3·1	0·0	2·2	0·0	2·0	0·0	3·5	0·0	2·1	0·0	2·0	0·0	3·4	0·0

UPF contributed significantly to the intake mentioned above of unhealthy nutrients. Compared to the fraction of the diet concerning non-UPF, the fraction referring to UPF has 1·6 to 1·8 more energy/g, 1·5 to 2 more *trans*-fat, 2·1 to 4·2 more added sugar, 3·3 to 5·3 more Na, depending on the age group. In addition, the UPF fraction was two times lower in fibre, proteins and K content. Non-processed and ultra-processed fractions have similar fat and saturated fat contents, especially in adolescents and women. Regarding saturated fats, its primary sources were both UPF and non-UPF because of the high consumption of animal products in Argentina, particularly red meat (Table 2).

### Nutrient profile and healthy food consumption according to UPF intake

An interesting pattern emerged when the population was classified according to their consumption of UPF and adjusted by sociodemographic variables. Table 3 presents the nutritional dietary profile indicators for the five strata of the population corresponding to increasing quintiles in energy contribution from UPF. Besides UPF being an important source of free sugar in all the analysed groups, a positive association between UPF consumption and free sugar intake was found only in children and adolescent females (*r* = 0·47 and 0·34, *P* < 0·001). While UPF did not contribute significantly to the intake of *trans*-fat, higher consumption of UPF was also positively associated with *trans*-fat intake in the three groups (*r* = 0·40, 0·46 and 0·51, *P* < 0·001). Na, total fat and saturated fat also increased with higher consumption of UPF, but the association was weaker (*r* < 0·30, *P* < 0·001; see Table 3). The same trend was observed for fibre, protein and K (*r* < -0·35, *P* < 0·001; Table 3 for more details). Also, we found a negative association between the consumption of UPF and fresh and whole foods (*r* < -0·30, *P* < 0·001), showing a potential displacement effect. This result was particularly evident in the highest quintile of UPF consumption, in which a deficient intake of fresh fruits was observed in all age groups analysed.

**Table 3:** Indicators of diet quality across quintiles of the dietary share of ultra-processed foods. Argentinian children and women population (2005)

Indicators**	Children aged 2–5 years							Adolescent aged 10–19 years							Women aged 20–49 years						
	Quintiles (Q)*** of the dietary share of ultra-processed foods (% of total energy)					Standardised regression coefficients†		Quintiles (Q)*** of the dietary share of ultra-processed foods (% of total energy)					Standardised regression coefficients†		Quintiles (Q)*** of the dietary share of ultra-processed foods (% of total energy)					Standardised regression coefficients†	
	Q1	Q2	Q3	Q4	Q5	Crude	Adjusted‡	Q1	Q2	Q3	Q4	Q5	Crude	Adjusted‡	Q1	Q2	Q3	Q4	Q5	Crude	Adjusted‡
Fresh vegetables (g/2000 kcal)	72·9	65·9	60·8	55·6	47·7	−0·11*	−0·12*	107·9	96·9	82·2	77·3	69·6	−0·11*	−0·16*	149·1	115·8	130·7	139·0	99·7	−0·05*	−0·07*
Fresh fruits (g/2000 kcal)	96·7	127·8	115·3	100·0	78·2	−0·07*	−0·11*	113·6	97·4	108·9	79·2	46·0	−0·14*	−0·19*	90·5	90·6	117·8	109·6	92·7	0·00*	−0·05*
Legumes (g/2000 kcal)	6·6	3·3	4·8	3·6	1·7	−0·06*	−0·05*	6·1	3·9	5·4	5·0	3·3	−0·02*	−0·05*	11·2	5·9	7·9	6·6	1·8	−0·07*	−0·06*
Nuts and seeds (g/2000 kcal)	0·3	0·1	0·1	0·2	0·3	−0·01*	−0·01*	0·5	1·2	1·1	0·8	1·4	0·05*	0·05*	0·1	0·4	0·4	0·6	0·9	0·05*	0·03*
Wholegrains (g/2000 kcal)	3·8	2·1	2·6	1·8	2·4	−0·02*	−0·03*	1·1	2·3	3·9	1·7	2·4	0·02*	0·01*	4·6	4·6	7·6	8·0	8·1	0·05*	0·02*
Fresh vegetables (g/2000 kcal)	72·9	65·9	60·8	55·6	47·7	−0·11*	−0·12*	107·9	96·9	82·2	77·3	69·6	−0·11*	−0·16*	149·1	115·8	130·7	139·0	99·7	−0·05*	−0·07*
% of total energy intake from:																					
Protein	14·8	15·1	14·6	14·2	12·6	−0·21*	−0·30*	14·2	14·6	14·0	13·7	12·2	−0·17*	−0·26*	14·3	14·6	15·5	14·8	13·1	−0·07*	−0·16*
Total carbohydrates	57·4	53·8	53·4	53·0	54·4	−0·10*	0·01*	60·0	55·4	53·8	53·6	52·2	−0·20*	−0·07*	58·3	54·4	51·7	50·4	50·8	−0·19*	−0·07*
Free sugar	12·9	15·6	18·4	19·9	23·3	0·43*	0·47*	14·2	14·9	18·4	19·1	21·3	0·28*	0·34*	18·6	17·6	17·7	18·0	18·0	−0·01*	0·08*
Total fat	27·7	31·1	32·0	32·8	32·9	0·20*	0·11*	25·7	30·0	32·1	32·7	35·5	0·29*	0·19*	27·3	30·6	32·3	34·1	35·6	0·24*	0·15*
Saturated fat	10·5	12·1	12·6	12·8	12·7	0·18*	0·07*	7·6	9·9	10·7	10·9	11·8	0·31*	0·22*	8·4	9·7	10·5	11·6	12·0	0·28*	0·18*
*Trans*-fat	0·7	0·9	1·0	1·2	1·3	0·43*	0·40*	0·5	0·7	0·9	1·0	1·3	0·49*	0·46*	0·5	0·7	0·8	1·0	1·3	0·54*	0·51*
Na (mg/d)	790	962	1070	1227	1404	0·30*	0·25*	857	1106	1263	1586	1753	0·30*	0·26*	714	1018	1148	1325	1592	0·30*	0·25*
Na (mg/2000 kcal)	1128	1211	1321	1416	1528	0·21*	0·17*	1080	1231	1211	1552	1546	0·22*	0·17*	1031	1226	1283	1502	1808	0·28*	0·24*
K (mg/d)	1827	2037	1993	1990	1801	−0·02*	−0·09*	1702	1921	2096	1770	1728	−0·03*	−0·08*	1480	1662	1814	1770	1548	−0·03*	−0·05*
K (mg/2000 kcal)	2624	2637	2492	2353	2040	−0·26*	−0·33*	2323	2177	2049	1892	1654	−0·27*	−0·32*	2285	2099	2197	2141	1843	−0·11*	−0·16*
Na:K ratio	0·5	0·5	0·6	0·7	0·8	0·33*	0·34*	0·5	0·7	0·7	1·0	1·2	0·30*	0·28*	0·5	0·7	0·7	0·9	1·2	0·33*	0·34*
Fibre density (g/2000 kcal)	11·7	10·8	10·2	9·4	8·9	−0·18*	−0·17*	14·1	12·3	12·0	11·6	10·3	−0·18*	−0·21*	13·8	11·8	13·0	13·1	13·2	0·00*	−0·02*
Energy density in solid foods (kcal/g)	2·0	2·0	2·1	2·1	2·2	0·14*	0·16*	2·0	2·1	2·2	2·2	2·5	0·30*	0·34*	2·1	2·1	2·1	2·1	2·3	0·08*	0·14*

* *P* value <0·001.

† Obtained by regressing indicators on quintiles of the dietary share of ultra-processed foods.

‡ Adjusted for sociodemographic variables (age, sex in children aged 2–5 years, geographic region, years of schooling of head of family in children and women aged 10–19 years, years of schooling of women aged 20–49 years, unsatisfaction of basic needs, income, income level (quintile)).

** Mean values.

*** Quintiles of energy from UPF (% kcal). In children aged 2–5 years (Q1: 0–9·3 %; Q2: 9·4–20·6 %; Q3: 20·7–31·1 %; Q4: 31·2–43·8 %; Q5: 43·9–100 %). In women aged 10–19 years (Q1: 0–8·3 %; Q2: 8·4–20·8 %; Q3: 20·9–32·8 %; Q4: 32·9–47·0 %; Q5: 47·1–100 %). In women aged 20–49 years (Q1: 0–5·4 %; Q2: 5·5–15·9 %; Q3: 16·0–27·1 %; Q4: 27·2–41·7 %; Q5: 41·8–100 %).

## Discussion

Our results showed that in 2005, the UPF supplied more than a quarter of total daily energy in children, adolescent females and adult females in Argentina. Across all age groups, the major contributors to UPF consumption were cookies and pastries (about 6·0–7·0 %), soft drinks (about 2·7–3·7 %), candies (about 1·8–4·6 %), and juices (about 1·3–1·7 %). We also found that UPF consumption was negatively associated with the intake of fresh vegetables, fresh fruits and legumes. It was the most important source of free sugar, Na and refined carbohydrates. It showed a significant positive association between the dietary share of UPF and the dietary content of NCD-promoting nutrients such as free sugars and total saturated and *trans*-fats, contributing significantly to the unhealthy profile of the Argentinian diet.

The estimated dietary share of UPF found here was similar to that reported in more recent studies for Mexico (29·8 % of total energy intake)^(15)^ and Chile (28·6 %)^(17)^ in the general population but higher than Brazil (21·5 %)^(11)^ and Colombia (15·9 %)^(14)^. Comparatively, these results are much lower than those found in high-income countries such as the USA (57·9 %)^(12)^, Canada (47·7 %)^(13)^ and Australia (42·0 %)^(16)^. However, studies that analysed the purchase of UPF have shown that it is likely that UPF consumption has increased during the last decade in Argentina, reaching nearly 30 % of daily energy^(31)^.

As was observed in our results, previous studies on food sales showed that^(29,31)^ cookies, pastries, crackers, soft drinks and juices, and sweets were the most consumed UPF in Argentina, a pattern of preference for UPF similar to that in other countries of the region^(29)^. In addition, we also found a deficient intake of fresh and whole foods such as fruits, vegetables, legumes, wholegrains, nuts and seeds in the analysed population, which contributed to undermining public health^(4)^. The observed increase in the consumption of UPF during the last decades in Argentina (including the ready-to-eat meals) has caused a displacement in the intake of healthy foods. For instance, in Argentina, fruits are usually consumed as a dessert, particularly after lunch, and in many cases is the only instance of fruit intake during the day. The inclusion of UPF desserts such as sweets, candies and ice cream could compete directly with the consumption of fruits. In addition, snacks also compete with the consumption of fruits in between main meals. In the same line, ready-to-eat meals displaced homemade meals’ with vegetables and other culinary ingredients. This dietary pattern is of concern given the high prevalence of childhood and adult overweight and obesity in Argentina and hypertension, coronary diseases, stroke, and type 2 diabetes in the general population^(43)^. In this sense, our findings provide helpful evidence for formulating national food policies to improve dietary quality.

Different strategies could be implemented in Argentina to decrease the consumption of UPF, from taxation and marketing controls to food subsidies, front-of-package labelling, and shifts in school foods and the school food environment^(44)^. These policies have already been shown to reduce the consumption of harmful products, such as tobacco and alcohol. Many countries have applied one or more of these initiatives^(45)^. Although each of these policies can have an effect on the consumption of UPF, the evidence suggests that the most promising approaches are those that comprise multiple coordinated policies. For instance, a 5 % increase in the price of soft drinks through taxation, implemented by the Government of Chile in 2014, reduced the consumption of 3 % of high-sugar beverages^(46)^. However, warning labelling in soft drinks decreased their consumption by 23 %^(47)^. National policies can substantially modify the food environment in which people purchase and consume foods and affect millions of people simultaneously and are crucial to preventing rapid increases in the intake of unhealthy food products and nutrition-related NCD^(48,49)^.

A bill is being intensely discussed in the Argentinian national congress for implementing a law that includes front-of-pack labelling of food and non-alcoholic beverages, regulation of marketing and advertising of unhealthy foods, and nutritional education in school environments. However, because of the low consumption of fruits, vegetables, wholegrains, legumes, nuts and seeds, food policies oriented towards increasing the intake of these food groups is also critical for dietary quality improvement^(4)^. Such policies should be designed in a holistic framework and then consider both supply- and demand-side interventions to create food environments that allow the population to make healthy choices^(50)^. Additionally, it might be convenient to include in the dietary guidelines recommendations according to the degree of food processing, including recommendations such as avoiding the consumption of UPF and increasing the consumption of natural foods, combinations of foods, dishes, and meals, and the social and cultural dimensions of dietary patterns in concordance with Brazil and Uruguay guidelines, neighbour countries with similar conditions to Argentina.

Potential limitations should be considered. The analysis was performed from one 24-h recall. However, the standardised methods and approach minimise possible error and bias, particularly for assessing population averages as focused on in the present study. In addition, although information indicative of food processing was collected, these data were missing for some food items and thus may have led to errors in food classification. As we had a conservative position regarding the classification of UPF (they were only classified as UPF when there was absolute certainty), exists a possible under-estimation in the estimation of consumption of these products. These data are more than a decade old. They correspond to the first national nutrition and health survey, the only one available so far, and it is expected that the new study in progress can update the landscape. At the same time, this analysis will provide a baseline and comparison point for future research. Though the addition of salt to meals is not included in the study. The high intake of Na in all fractions suggests that in addition to reducing the Na content of industrialised foods, it is necessary to strengthen campaigns to minimise the addition of salt to meals during their preparation or consumption at the domestic level.

Our study has several strengths. We studied a large, nationally representative sample of the infant and maternal Argentina population. This study is the first to evaluate the dietary contribution of UPF and the NCD-related nutrient profile of Argentinian diets providing updated and relevant results for informing the public health agenda. These may also serve as baseline results to measure the impact of a set of regulations being implemented by the Argentinian government aimed at improving diets.

## Conclusion

UPF consumption contributed to the total energy intake in Argentina’s children and women of reproductive age, reaching more than 25 %. In addition, the dietary share of UPF negatively affected the consumption of fresh and whole foods. It was positively associated with the intake of critical nutrients for NCD development, mainly free sugar.

Due to the high prevalence of overweight/obesity and other food-related NCD in Argentina, decreasing the consumption of UPF through food policies is urgent. However, these initiatives should be accompanied by different strategies that aim to improve dietary quality by increasing unprocessed or minimally processed foods consumption to guide the population to achieve healthy eating recommendations.

## Supplementary material

For supplementary material accompanying this paper visit https://doi.org/10.1017/S1368980022002543. click here to view supplementary material
