# Supplementary material for: Ultra-processed foods consumption and diet quality among preschool children and women of reproductive age from Argentina
Source: Public Health Nutr. 2022 Dec 16;26(11):2304–13. doi: 10.1017/S1368980022002543 (PMC10641612; doi:10.1017/S1368980022002543)
Supplement: Supplementary file 1 [file S1368980022002543sup001.docx]

**Supplemental material**

**Table 1.** Categorization of food and beverages into NOVA classification

| 1- Unprocessed or minimally processed foods | Vegetables | Chard |
| --- | --- | --- |
|  |  | Chicory |
|  |  | Red chili pepper / red bell pepper |
|  |  | Green chili pepper / green or yellow bell pepper |
|  |  | Artichoke |
|  |  | Celery |
|  |  | Fresh pea |
|  |  | Eggplant |
|  |  | Watercress |
|  |  | Broccoli |
|  |  | Bean sprouts |
|  |  | Onion |
|  |  | Green bean |
|  |  | Fresh corn |
|  |  | Cauliflower |
|  |  | Asparagus |
|  |  | Spinach |
|  |  | Fresh beans |
|  |  | Fennel |
|  |  | Fresh mushrooms |
|  |  | Lettuce |
|  |  | Cucumber |
|  |  | Leek |
|  |  | Radish |
|  |  | Radicheta |
|  |  | Beet |
|  |  | Brussels sprout |
|  |  | Cabbage |
|  |  | Fresh tomato |
|  |  | Carrot |
|  |  | Zucchini |
|  |  | Squash |
|  | Roots and tubers | Sweet potato |
|  |  | Cassava |
|  |  | Potato |
|  | Fruits | Pineapple |
|  |  | Banana |
|  |  | Fresh cherry (pulp and skin) |
|  |  | Raw prune |
|  |  | Fresh pulp plum |
|  |  | Fresh coconut |
|  |  | Damascus |
|  |  | Fresh peach |
|  |  | Raw dried peach with stone |
|  |  | Raw strawberry / pitanga / blackberry |
|  |  | Grenade |
|  |  | Guava |
|  |  | Kiwi / prickly pear |
|  |  | Fresh pulp lemon |
|  |  | Mamón |
|  |  | Tangerine |
|  |  | Mango |
|  |  | Apple with skin |
|  |  | Skinless apple |
|  |  | Fresh pulp melon |
|  |  | Raw fresh pulp quince |
|  |  | Orange |
|  |  | Nispero / guabirva |
|  |  | Avocado |
|  |  | Raisins |
|  |  | Pear |
|  |  | Grapefruit |
|  |  | Watermelon |
|  |  | Fresh grape |
|  | Cereals | Amaranth |
|  |  | White rice |
|  |  | Integral rice |
|  |  | Oats |
|  |  | Dry noodles |
|  |  | Algarrobo / Patay Flour |
|  |  | Rice flour |
|  |  | Barley flour |
|  |  | Rye flour |
|  |  | Cornmeal |
|  |  | Soybean flour |
|  |  | Wheat flour |
|  |  | Whole wheat flour |
|  |  | Whole grain corn |
|  |  | Raw Seed Quinoa / Flax Seed |
|  |  | Oat bran |
|  |  | Wheat bran |
|  |  | Semola - vitina - anchi |
|  |  | Tapioca (cassava flour) |
|  |  | Raw whole grain wheat |
|  | Legumes | Pea raw whole dried |
|  |  | Pea or wheat germ raw split dried seed |
|  |  | Garbanzo beans |
|  |  | Lentils |
|  |  | Beans |
|  |  | Soy beans |
|  | Nuts | Almond / sunflower seed |
|  |  | Hazelnut |
|  |  | Nut |
|  | Milk and plain yogurt | Goat milk whole |
|  |  | Cow's milk whole |
|  |  | Cow's milk whole with vitamins A and D |
|  |  | Cow's Milk Part Skim |
|  |  | Cow's Milk Part Skim with Vitamins A and D |
|  |  | Skim milk powder |
|  |  | Lactose-free milk powder with vitamins A and D |
|  |  | Whole milk powder |
|  |  | Whole milk powder fortified with iron (Maternal and infant plan and others) |
|  |  | Dehydrated fluid whole milk with vitamins A and D |
|  |  | Iron-fortified fluid whole milk |
|  |  | Part-skim milk Lactose-free fluid with vitamins A and D |
|  |  | Whole plain yogurt |
|  | Meat | Goat |
|  |  | Pork/ Capybara |
|  |  | Chinchulines |
|  |  | Rabbit |
|  |  | Lamb |
|  |  | Liver |
|  |  | Language |
|  |  | Sweetbread |
|  |  | Tripe |
|  |  | Duck |
|  |  | Chicken with skin |
|  |  | Chicken offal |
|  |  | Chicken without skin / Vizcacha / Jacare / Garza / Partridge / Charata (bird) / Lampalagua (snake) |
|  |  | Kidney |
|  |  | Brains |
|  |  | Udder |
|  |  | Beef, cuts with bone: empty roast minced meat common needle skirt alita dura matambre Guazuncho |
|  |  | Beef, boneless cuts: vacuum roast minced meat common needle skirt flank hard matambre Guazuncho |
|  |  | Beef, boneless cuts: loin ball buttock peceto paddle square rump |
|  | Fish and seafood | Squid |
|  |  | Mussel |
|  |  | Sea Fish average: White corvina, brool, hake, sole, catfish |
|  |  | River Fish average: Trout, palometa, surubí, dorado |
|  | Egg | Quail Egg |
|  |  | Egg white |
|  |  | Egg |
|  |  | Egg yolk |
| 2-Processed culinary ingredients | Table sugar | White sugar |
|  |  | Brown sugar |
|  | Animal fats | Milk cream |
|  |  | Pork fat |
|  |  | Beef fat |
|  |  | Butter |
|  | Plant oils | Edible oil blend |
|  |  | Canola oil |
|  |  | Sunflower oil |
|  |  | Corn oil |
|  |  | Olive oil |
|  |  | Soy oil |
|  |  | Oil Grape |
|  | Other processed culinary ingredient | Cornstarch |
|  |  | Honey / Arrope / Kero |
| 3- Processed foods | Breads (fresh unpackaged) | Country biscuit |
|  |  | Arabic bread |
|  |  | Creole bread / semita / tortillas / buns / Flour |
|  |  | Cornbread |
|  |  | Bread crumb |
|  |  | French bread |
|  |  | Bread crumbs |
|  | Cheese | Soft Cheeses: cuartirolo, double cream, Limburgues, mozzarella |
|  |  | Hard Cheeses: Goya, Parmesan, Provolone, Reggianito, Sardo, Sbrinz |
|  |  | Medium Hard Cheeses: Azul, Chubut, Fontina, Gruyère, Holland, Mar del Plata, Pategras, Machine cheese, Roquefort, Taif, Tandil |
|  |  | Ricotta |
|  | Ham and other salted, smoked or canned meat or fish | Anchovy in oil |
|  |  | Tuna in oil |
|  |  | Tuna in water |
|  |  | Canned mackerel |
|  |  | Chorizo |
|  |  | Cooked ham |
|  |  | Raw ham |
|  |  | Blood sausage |
|  |  | Bacon |
|  |  | Sardines in oil |
|  | Vegetables, fruits and other plant foods preserved in brine or syrup | Pickled pulp green olives |
|  |  | Canned pineapple |
|  |  | Canned fresh peas |
|  |  | Canned corn kernels |
|  |  | Natural packed peach pulp |
|  |  | Canned mushrooms |
|  |  | Canned palm hearts |
|  |  | Canned natural pear |
|  |  | Canned tomato |
|  | Other processed foods | Chipa |
|  |  | Churros |
|  |  | Sweet Batata |
|  |  | Quince sweet |
|  |  | Quince jelly |
|  |  | Fruit jam |
|  |  | Potato Gnocchi |
|  |  | Fried cake |
|  |  | Salty roasted peanuts |
|  | Alcoholic beverages | Beer with alcohol |
|  |  | Alcohol-free beer |
|  |  | Wine |
| 4- Ultra-processed foods | Soft drinks, carbonated | Soft drinks |
|  |  | Diet soft drinks |
|  | Fruit drinks/sweetened water | Juice |
|  |  | Powdered juices |
|  |  | Diet juice powder |
|  |  | Liquid concentrated soft drink / frozen juices in sachet / Achilate |
|  | Other non alcoholic beverages | Bitter serrano |
|  |  | Bitter serrano diet |
|  |  | Soy-based drinks |
|  |  | Light soy-based drinks |
|  | Breads (packaged) | Pizza mass |
|  |  | Rye bread |
|  |  | Gluten bread |
|  |  | Bran bread |
|  |  | Viena bread |
|  |  | Lactal bread |
|  |  | Lactal bread with bran |
|  |  | Unleavened black bread |
|  | Cakes, cookies and pies | Grease cakes / Mantecado / Jachaleras pancakes |
|  |  | Bay Biscuit type cakes / anise rolls |
|  |  | Canale type cakes |
|  |  | Pastries (Facturas) |
|  |  | Croissants |
|  |  | Stuffed sweet cookies |
|  |  | Manon type cookies |
|  |  | Wafers / Cucurucho / Cubanito without filling |
|  |  | Vanillas |
|  | Crackers (refined and whole) | Marinera biscuit |
|  |  | Unsalted water crackers |
|  |  | Rice crackers |
|  |  | Wholegrain crackers without salt |
|  |  | Whole grain cookies |
|  |  | Express type cookies |
|  |  | Breadsticks |
|  | Breakfast cereals and cereal bars | Cereal bars / nougat |
|  |  | Fortified Infant Cereal |
|  |  | Sugary cereal flakes / Tutuca / Puflito |
|  |  | Big wave |
|  |  | All bran type wheat bran |
|  | Doughs and pasta | Cappelettis |
|  |  | Raw fresh noodles |
|  |  | Tart dough or empanadas |
|  |  | Fresh ravioli |
|  | Milk based drink and yogurt | Chocolate milk |
|  |  | Cindor Chocolate Milk |
|  |  | Skim Probiotics |
|  |  | Whole probiotics |
|  |  | Low-fat yogurt |
|  |  | Drinkable skim yogurt |
|  |  | Whole drinkable flavored yogurt |
|  |  | Whole flavored yogurt |
|  | Milk desserts | Ready-to-eat diet flan |
|  |  | Flan ready to eat |
|  |  | Drinkable milk desserts (Danonino type) |
|  |  | Ready-to-eat milk desserts (Danonino type) |
|  |  | Ready-to-eat milk desserts (Serenito type) |
|  |  | Diet ready-to-eat milk desserts (type Ser) |
|  | Infant formula | Powdered starter formula |
|  |  | Fluid starter formula |
|  |  | Follow-up formula powdered milk |
|  |  | Fluid follow-up formula |
|  | Cheese (spreadable and melted) | Molten cheese |
|  |  | Whole spreadable cream cheese |
|  |  | Skim cheese spread |
|  |  | Spread semi-skim cheese |
|  | Reconstituted meat | Mortadella |
|  |  | Pate / mince |
|  |  | Headcheese |
|  |  | Salami |
|  |  | Vienna sausage |
|  |  | Barbecue sausage |
|  | *Dulce de leche* | Caramel sauce |
|  | Cocoa and milk flavorings | Cocoa powder |
|  |  | Chocolate flavoring |
|  | Sweets and candies | Chocolate Alfajor / Rhodesia |
|  |  | Alfajor of dulce de leche / alfajor of cornstarch |
|  |  | Filled candy |
|  |  | Hard candies |
|  |  | Diet hard candies |
|  |  | Chewy candies |
|  |  | Chocolate for cup / butter |
|  |  | Chocolate bar |
|  | Desserts | Powdered flan |
|  |  | Prepared dietary gelatin |
|  |  | Prepared gelatin |
|  |  | Water ice cream |
|  |  | Ice cream |
|  |  | Powder to prepare fortified peach dessert |
|  |  | Powder to prepare fortified vanilla dessert |
|  |  | Powder to prepare fortified chocolate dessert |
|  | Margarine | 100% vegetable margarine (in pot and in bread) |
|  |  | Spread reduced calorie margarine |
|  | Sauces, dressings and gravies | Ketchup |
|  |  | Mayonnaise |
|  |  | Soy mayonnaise |
|  |  | Mayonnaise reduced in calories |
|  |  | Mustard |
|  |  | Golf sauce |
|  | Salty snacks | Chizitos |
|  |  | Sticks |
|  |  | Chips |
|  | Sandwiches & hamburgers on bun | Industrialized beef burgers |
|  |  | Industrialized Chicken Burgers |
|  | Instant meals | Portuguese rice |
|  |  | Fortified rice a la yaya |
|  |  | Fortified spring rice |
|  |  | Noodles with sauce |
|  |  | Campero stew |
|  |  | Lents' stew |
|  |  | Creole stew |
|  |  | Fortified Manchega lentils |
|  |  | Soy Milanesa |
|  |  | Instant mashed potatoes |
|  | Instant and canned soups | Broths in cube |
|  |  | Diet powder bouillon |
|  |  | Instant diet cream soup |
|  |  | Instant cream soup |
|  | Other ultra-processed foods | Creamy canned choco |
|  |  | Diet packaged peach |
|  |  | Canned natural pear diet |

**Table 2.** Categorization of food and beverages into categories of healthy foods

| Fresh vegetables | Chard |
| --- | --- |
|  | Chicory |
|  | Red chili pepper / red bell pepper |
|  | Green chili pepper / green or yellow bell pepper |
|  | Artichoke |
|  | Celery |
|  | Fresh pea |
|  | Eggplant |
|  | Watercress |
|  | Broccoli |
|  | Bean sprouts |
|  | Onion |
|  | Green bean |
|  | Fresh corn |
|  | Cauliflower |
|  | Asparagus |
|  | Spinach |
|  | Fresh beans |
|  | Fennel |
|  | Fresh mushrooms |
|  | Lettuce |
|  | Cucumber |
|  | Leek |
|  | Radish |
|  | Radicheta |
|  | Beet |
|  | Brussels sprout |
|  | Cabbage |
|  | Fresh tomato |
|  | Carrot |
|  | Zucchini |
|  | Squash |
| Fresh fruits | Pineapple |
|  | Banana |
|  | Fresh cherry (pulp and skin) |
|  | Raw prune |
|  | Fresh pulp plum |
|  | Fresh coconut |
|  | Damascus |
|  | Fresh peach |
|  | Raw dried peach with stone |
|  | Raw strawberry / pitanga / blackberry |
|  | Grenade |
|  | Guava |
|  | Kiwi / prickly pear |
|  | Fresh pulp lemon |
|  | Mamón |
|  | Tangerine |
|  | Mango |
|  | Apple with skin |
|  | Skinless apple |
|  | Fresh pulp melon |
|  | Raw fresh pulp quince |
|  | Orange |
|  | Nispero / guabirva |
|  | Avocado |
|  | Pear |
|  | Grapefruit |
|  | Watermelon |
|  | Fresh grape |
|  | Acerola |
|  | Cayota o Alcayota |
| Legumes | Pea raw whole dried |
|  | Pea or wheat germ raw split dried seed |
|  | Garbanzo beans |
|  | Algarrobo / Patay Flour |
|  | Soybean flour |
|  | Lentils |
|  | Beans |
|  | Soy beans |
|  | Soy Milanesa |
|  | Canned fresh peas |
| Nuts and seeds | Almond / sunflower seed |
|  | Hazelnut |
|  | Salted Roasted Peanuts |
|  | Nut |
|  | Pistachios |
| Whole grains | Amaranth |
|  | Integral rice |
|  | Oats |
|  | Wholegrain crackers |
|  | Rye flour |
|  | Barley flour |
|  | Whole wheat flour |
|  | Whole grain corn |
|  | Rye bread |
|  | Unleavened black bread |
|  | Raw Seed Quinoa / Flax Seed |
|  | Oat bran |
|  | Wheat bran |
|  | All bran type wheat bran |
|  | Raw whole grain wheat |
|  | Wholegrain crackers without salt |
